# Clinical Markers of Crohn’s Disease Severity and Their Association With Opiate Use

**DOI:** 10.14740/jocmr1969w

**Published:** 2014-10-16

**Authors:** Mary Cheung, Sundas Khan, Meredith Akerman, Chun Kit Hung, Kaitlyn Vennard, Nicholas Hristis, Keith Sultan

**Affiliations:** a Department of Medicine, Division of Gastroenterology, Hofstra North Shore-LIJ School of Medicine, Manhasset, NY 11030, USA; b Department of Medicine, Hofstra North Shore-LIJ School of Medicine, Manhasset, NY 11030, USA; c The Feinstein Institute for Medical Research, Manhasset, NY 11030, USA

**Keywords:** Crohn’s disease, Opiates, Fistulas, Smoking, Inflammatory bowel disease

## Abstract

**Background:**

The safety of opiate use for patients with Crohn’s disease (CD) has long been a concern. The recent Crohn’s therapy, resource, evaluation, and assessment tool (TREAT) registry update has added to these concerns by demonstrating an association of opiate use with an increased risk of infection and death in CD. While the association is clear, the relationship of opiates to these negative outcomes is not. It is unknown whether opiates are a contributing factor to these negative outcomes or if their use is merely a marker of more severe disease. We hypothesized that opiate use is not harmful in CD but is a marker of disease severity and would be associated with commonly accepted clinical markers of severe CD such as early age at CD onset, disease duration, small intestinal involvement, a history of fistula or stricture, and lower quality of life (QOL) scores.

**Methods:**

Data on CD history including pain medication usage were obtained from an interviewer directed survey of patients admitted to two tertiary care hospitals over a 2-year period. CD as the primary admitting diagnosis was not required. Active opiate use was defined by usage within the past month prior to admission.

**Results:**

A total of 133 patients were approached to participate, of whom 108 consented to the survey, and 51 were active opiate users. Opiate using CD patients were more commonly smokers (22% vs. 3.45%, P < 0.010), had fistulas (40% vs. 22.4%, P < 0.048) and had a poorer quality of life score by short form inflammatory bowel disease questionnaire (mean 3.80 vs. 4.34, P < 0.036) than non-opiate users. No difference was found between opiate users and non-users for age of diagnosis, disease duration, or a history of strictures.

**Conclusions:**

The study findings demonstrate that opiate use in CD is associated with markers of disease severity including fistulas, smoking, and lower QOL scores. The findings suggest that opiates may not be directly harmful to patients with CD, but may merely be another marker of disease severity. However, given opiates unproven benefits for long term CD pain control and risk of dependence, caution should still be exercised in their use.

## Introduction

Effective and safe pain management for patients with Crohn’s disease (CD) has long been a source of controversy. Non-steroidal anti-inflammatory medications (NSAIDs) may pose a risk of CD exacerbation [1], and though opiate pain medications may be effective for acute pain, their usefulness for chronic CD related pain is unproven. Additionally, opiates pose a risk of addiction [2] and in the case of chronic abdominal pain may even cause a paradoxical worsening of pain symptoms termed narcotic bowel syndrome [3]. There are also concerns that opiates may mask CD complaints, thus altering management and outcomes [4]. Recent data from the Crohn’s therapy, resource, evaluation, and assessment tool (TREAT) registry update have added to these concerns by noting an association between opiate use and the development of infections and an increased risk of death [5].

Prior studies have examined factors associated with opiate use in CD. Cross et al reviewed 291 CD patients over a 5-year period, of which 38 were chronic opiate users [6]. The authors found opiate use to be associated with disease activity, disability, female gender, polypharmacy, smoking, and longer disease durations. In another analysis, Hanson et al matched 100 opiate using mixed inflammatory bowel disease (IBD) patients with 100 non-opiate using controls [7]. An association was found with female gender, moderate to severe pain, depression, anxiety, history of abuse (sexual, emotional, or physical) and for substance abuse apart from alcohol. Earlier work by Edwards et al also noted a strong association between opiate use and psychiatric disorders [8].

While each of these studies provides important insights into associated co-factors possibly contributing to opiate use, the question remains: are opiates a marker of disease severity and the associated negative outcomes, or a cause of these negative outcomes? Both Cross and Hanson found a relationship between opiate use and smoking, which is known to be associated with worsened disease severity and poorer outcomes [9]. Other clinical factors commonly associated with severe CD include early age at CD onset, disease duration, small intestinal involvement, and a history of fistula or stricture [10-14] and quality of life (QOL) scores. We hypothesized that opiates are not harmful to patients with CD, but rather are a marker of more severe CD and would be associated with these clinical factors.

## Materials and Methods

Data were obtained from an interviewer directed survey of CD patients upon admission to two acute care, non-referral center hospitals over a 2-year period. Patients were identified by an electronic medical record search of admission history documents for the keyword “Crohn’s” or “Crohn”. Following identification as a potential study candidate, patients themselves needed to confirm a diagnosis of CD. CD exacerbation as the primary admitting diagnosis was not required. CD history obtained included age of diagnosis, disease duration, disease location, history of fistula, history of stricture, smoking status, opiate use, and QOL with the short form inflammatory bowel disease questionnaire (SIBDQ). Pain assessment was obtained using the Harvey Bradshaw index (HBI) pain subscore question 2 (HBI Q2) along with SIBDQ pain subscore question 4 (SIBDQ Q4). Irritable bowel syndrome (IBS) was diagnosed by ROME III criteria. Current opiate use was defined as usage within the month prior to hospital admission. The survey was comprised of closed ended questions administered at the bedside by either the division research coordinator or a research volunteer trained by the coordinator. The study including the survey was approved by our institutional review board.

### Statistical methods

Current opiate users and non-opiate users were compared using the Chi-square test or Fisher’s exact test, as deemed appropriate, for categorical variables and the two-sample *t*-test was used to compare the two groups for continuous variables. Disease duration was analyzed by applying standard methods of survival analysis, i.e., computing the Kaplan-Meier product limit curves, where group (opiate user vs. non-opiate user) was the stratification variable. No data were considered censored. The two groups were compared using the log-rank test. The median disease duration and corresponding 95% confidence intervals for each group were obtained from the Kaplan-Meier/product-limit estimates.

A result was considered statistically significant at the P < 0.05 level of significance. All analyses were performed using SAS version 9.3 (SAS Institute Inc., Cary, NC, USA).

## Results

One hundred thirty-three CD patients were approached with 108 patients consenting to the questionnaire. Sixty-seven patients (62%) were male and 51 (47%) were current opiate users. All opiate use was for pain control, i.e. none for management of diarrhea. There was no significant difference between opiate users and non-users for any demographic variable (Table 1).

**Table 1:** Patient Demographics

	Opiate users (n = 50)	Non-opiate users (n = 58)	P-value
Age	48.46 ± 15.99	52.28 ± 18.96	0.265
Gender (males)	30 (60.00%)	37 (63.79%)	0.686
Age of diagnosis	29.41 ± 15.68	33.42 ± 16.78	0.213
Disease duration (years)	14.5 (12.0, 24.0)	15.0 (10.0, 21.0)	0.769

Current tobacco use was more common among current opiate users vs. non-opiate users (22% vs. 3.45%, P < 0.010) (Table 2). A history of fistula was more common in opiate vs. non-opiate users (40% vs. 22.4%, P < 0.048). Enteroenteric fistulas were the only fistula subtype associated with opiate use (22% vs. 6.9%, P < 0.024). A univariate logistic regression model was run for the entire group of 108 patients to examine for any relationship between smoking status and fistulas. There was no evidence of active smoking status predicting fistulas (P < 0.6948). A univariate logistic regression model was also performed for the 51 current opiate users. Again, there was no evidence of active smoking status predicting fistulas (P < 0.9154).

**Table 2:** Clinical Characteristics and History

	Opiate users (n = 50)	Non-opiate users (n = 58)	P-value
SIBDQ	3.80 ± 1.21	4.34 ± 1.35	0.036
IBS diagnosis	10 (20.00%)	8 (13.79%)	0.388
History of fistula	20 (40.00%)	13 (22.41%)	0.048
Enterocutaneous	4 (8.00%)	4 (6.90%)	1.000
Enteroenteric	11 (22.00%)	4 (6.90%)	0.024
Entervesicular	1 (2.00%)	0 (0.00%)	0.463
Rectovaginal	1 (2.00%)	3 (5.17%)	0.622
Perianal	9 (18.00%)	12 (20.69%)	0.725
Any surgical history	33 (66.00%)	36 (62.07%)	0.672
History of abscess drain	12 (24.00%)	15 (25.86%)	0.824
History of bowel resection	27 (54.00%)	21 (36.21%)	0.064
Ostomy	9 (18.00%)	12 (20.69%)	0.725
Stricture	8 (16.00%)	10 (17.24%)	0.863
Tobacco			
Current	11 (22.00%)	2 (3.45%)	0.010
Past	18 (36.00%)	22 (37.93%)	
Never	21(42.00%)	34 (58.62%)	
Past cocaine	1 (2.00%)	1 (1.72%)	1.000
Past marijuana	6 (12.00%)	7 (12.07%)	0.991
Alcohol			
Daily	3 (5.26%)	2 (4.00%)	0.479
Weekly	10 (17.54%)	8 (16.00%)	
Monthly	10 (17.54%)	9 (18.00%)	
Few times/year	24 (42.11%)	15 (30.00%)	
Never	10 (17.54%)	16 (32.00%)	

Strictures (76% vs. 68.97%, P < 0.416) and small bowel disease (40% vs. 31%, P < 0.331) were not associated with opiate use. There was a non-significant trend towards a history of bowel resection among the opiate using patients (54% vs. 36.2%, P < 0.064). There was no difference between opiate users vs. non-opiate users for age of diagnosis (29.4 vs. 33.4 years, P < 0.213) or disease duration (median 14.5 years vs. 15.0 years, P < 0.769). Opiate use was not found to be associated with alcohol, cocaine, or marijuana use. Abdominal pain by HBI Q2 score was similar between opiate and non-opiate users (mean 2.11 vs. 1.82, P < 0.243) as was abdominal pain by IBDQ Q4 score (mean 3.67 vs. 4.13, P = 0.297), although QOL was significantly lower (mean 3.80 vs. 4.34, P < 0.036) for opiate users.

## Discussion

In this non-referral center based study of hospitalized patients with CD, we have confirmed prior reports showing an association between opiate use and active smoking [6, 7], and have observed a novel association of opiates with a history of fistulas. Though it has been suggested that smoking itself might be associated with fistulas in CD [15], there are little data to support this, and our univariate logistic regression analysis shows no evidence that this was the case for our patients specifically. Fistulas are among the most objective findings of severe CD. While opiates may mask or even mimic symptoms of CD, there is no known mechanism by which they cause fistulas, suggesting opiates as a response to disease severity in our patient population rather than a cause of disease severity. Additionally we noted that QOL was lower among opiate users. While QOL determined by questionnaire is not as objective a finding as fistula, it too correlates with disease severity and suggests an increased disease burden in the opiate users [16, 17].

The main limitation of our study, common to any survey based investigation, is the dependence upon patient recall. While “objective” disease findings for details such as age of diagnosis are less likely to be affected by the survey format, it would be reasonable to question the accuracy of patient recall of details such as disease location and history of fistula or stricture without imaging, endoscopic or surgical reports for confirmation. Though the patients were seen in a tertiary care setting, the hospitals involved are not part of a closed system. As such, the vast majority of patients interviewed were followed by community gastroenterologists, with their outpatient records such as endoscopic reports and imaging inaccessible to review by the study team. Although our survey was not validated against patient medical records, recent experience with patient self reporting of IBD survey data as part of the Crohn’s and Colitis Foundation Partners (CCFA) study has in fact shown a good correlation between patient knowledge and disease history [18].

Other potential limitations of the study include the method and location of recruitment. Identification of study candidates required documentation of CD within the admission history of the hospitals’ electronic medical record. Particularly for patients admitted without active CD related complaints, it is likely that some patients did not have their CD history documented and were thus missed. Also, while our study benefits from the non-referral based setting of patient recruitment, the inpatient location of patient recruitment might bias towards individuals with more severe disease overall. While this had the benefit of providing a population of CD patients with higher rates of opiate use to analyze than seen by prior investigators, it is possible that this may not reflect the broader CD population.

In conclusion, we report the new finding of an association between opiate use and a history of fistula among patients with CD. We also provide further evidence of an association between smoking and opiate use in this population as well as poorer QOL scores among opiate users. Both fistula and smoking have been previously associated with more severe CD, suggesting that the use of opiates in this population may be a response to disease severity rather than a cause of disease severity. Even if we accept the conclusion that opiates are not directly harmful to patients with CD, this should not be viewed as an immediate license for increased usage. The risk of addiction or dependence as well as the unproven benefits of opiates for long term control of CD pain should be enough to recommend for continued caution with their use.

## References

[1] Singh S, Graff LA, Bernstein CN (2009). Do NSAIDs, antibiotics, infections, or stress trigger flares in IBD?. Am J Gastroenterol.

[2] Kaplan MA, Korelitz BI (1988). Narcotic dependence in inflammatory bowel disease. J Clin Gastroenterol.

[3] Grunkemeier DM, Cassara JE, Dalton CB, Drossman DA (2007). The narcotic bowel syndrome: clinical features, pathophysiology, and management. Clin Gastroenterol Hepatol.

[4] Jackson WE, Rizk M, Lashner BA (2013). Avoiding narcotics in Crohn's disease. J Clin Gastroenterol.

[5] Lichtenstein GR, Rutgeerts P, Sandborn WJ, Sands BE, Diamond RH, Blank M, Montello J (2012). A pooled analysis of infections, malignancy, and mortality in infliximab- and immunomodulator-treated adult patients with inflammatory bowel disease. Am J Gastroenterol.

[6] Cross RK, Wilson KT, Binion DG (2005). Narcotic use in patients with Crohn's disease. Am J Gastroenterol.

[7] Hanson KA, Loftus EV, Jr., Harmsen WS, Diehl NN, Zinsmeister AR, Sandborn WJ (2009). Clinical features and outcome of patients with inflammatory bowel disease who use narcotics: a case-control study. Inflamm Bowel Dis.

[8] Edwards JT, Radford-Smith GL, Florin TH (2001). Chronic narcotic use in inflammatory bowel disease patients: prevalence and clinical characteristics. J Gastroenterol Hepatol.

[9] Nunes T, Etchevers MJ, Domenech E, Garcia-Sanchez V, Ber Y, Penalva M, Merino O (2013). Smoking does influence disease behaviour and impacts the need for therapy in Crohn's disease in the biologic era. Aliment Pharmacol Ther.

[10] Cosnes J, Cattan S, Blain A, Beaugerie L, Carbonnel F, Parc R, Gendre JP (2002). Long-term evolution of disease behavior of Crohn's disease. Inflamm Bowel Dis.

[11] Zallot C, Peyrin-Biroulet L (2012). Clinical risk factors for complicated disease: how reliable are they?. Dig Dis.

[12] Beaugerie L, Seksik P, Nion-Larmurier I, Gendre JP, Cosnes J (2006). Predictors of Crohn's disease. Gastroenterology.

[13] Loly C, Belaiche J, Louis E (2008). Predictors of severe Crohn's disease. Scand J Gastroenterol.

[14] Lakatos PL, Czegledi Z, Szamosi T, Banai J, David G, Zsigmond F, Pandur T (2009). Perianal disease, small bowel disease, smoking, prior steroid or early azathioprine/biological therapy are predictors of disease behavior change in patients with Crohn's disease. World J Gastroenterol.

[15] Picco MF, Bayless TM (2003). Tobacco consumption and disease duration are associated with fistulizing and stricturing behaviors in the first 8 years of Crohn's disease. Am J Gastroenterol.

[16] Guyatt G, Mitchell A, Irvine EJ, Singer J, Williams N, Goodacre R, Tompkins C (1989). A new measure of health status for clinical trials in inflammatory bowel disease. Gastroenterology.

[17] Irvine EJ, Zhou Q, Thompson AK (1996). The Short Inflammatory Bowel Disease Questionnaire: a quality of life instrument for community physicians managing inflammatory bowel disease. CCRPT Investigators. Canadian Crohn's Relapse Prevention Trial.. Am J Gastroenterol.

[18] Randell RL, Long MD, Cook SF, Wrennall CE, Chen W, Martin CF, Anton K (2014). Validation of an internet-based cohort of inflammatory bowel disease (CCFA partners). Inflamm Bowel Dis.

